# Emergency Department Diagnosis of Multiple Mycotic Aneurysms in an HIV-Positive Patient Using Point-of-Care Ultrasound

**DOI:** 10.7759/cureus.21620

**Published:** 2022-01-26

**Authors:** Piyachat Sasipattarapong, Emily Johnson, Samuel Beckerman, Dana Sajed

**Affiliations:** 1 Emergency Medicine, University of Southern California, Los Angeles, USA

**Keywords:** emergency, ultrasound, point-of-care ultrasonography, hiv, pericarditis, mycotic aortic aneurysm

## Abstract

We report the case of a transgender woman with HIV (CD4 349), shortness of breath, and chest pain, who was found to have multiple mycotic aortic aneurysms by point-of-care ultrasound (PoCUS). This report highlights the utility of point-of-care ultrasonography in the diagnosis and management of this rare clinical entity.Multiple mycotic aortic aneurysms and purulent pericarditis are uncommon. They have high morbidity and mortality and are associated with immunocompromised states (e.g., HIV). Diagnosis of the mycotic aneurysm, and its precursor, infectious aortitis, can be challenging, and delays in care can lead to poor outcomes. Often, as described in this case report, making the diagnosis requires a high clinical suspicion, multiple imaging modalities, and laboratory studies.

## Introduction

Mycotic aortic aneurysm, infectious aortitis, and purulent pericarditis are rare clinical entities with established links to HIV infection and immunocompromised states [1,2]. HIV infection may predispose patients to mycotic aneurysms through different mechanisms, including underlying inflammatory states, as well as opportunistic infections that lead to the fragility in the intimal wall [3-6]. Rates of rupture are estimated at 85% and in-hospital mortality as high as 36% [7]. Unfortunately, diagnosis of the mycotic aneurysm, and its precursor, infectious aortitis, can be difficult. Often definitive diagnosis requires a high clinical suspicion, multiple imaging modalities, and microbiology and pathology studies [8-11]. Here, we present the case of an HIV-positive transgender female with an innominate artery mycotic aneurysm diagnosed with computed tomography (CT), as well as mycotic abdominal aortic aneurysms (AAA) first identified using point-of-care-ultrasound (PoCUS) in the Emergency Department (ED). This case highlights the importance of utilizing PoCUS to diagnose vascular emergencies and improve patient management.

## Case presentation

A 53-year-old male-to-female transgender patient with HIV on antiretroviral therapy was brought in by ambulance with a chief complaint of sudden onset pleuritic chest pain and shortness of breath. The patient reported that her pain started while she was gardening, approximately 18 hours before arrival in the ED. She described the pain as sharp and substernal. The pain did not radiate, nor was it positional or associated with eating. The patient denied nausea, vomiting, diaphoresis, or recent trauma. She denied fever, cough, hematemesis, history of venous thromboembolism, recent surgery, immobilization, or current hormone use. She also denied any rash, diarrhea, melena, or dysuria. The patient endorsed a history of polysubstance abuse and recent use of methamphetamine. Her CD4 count measured during this admission was 349 cells/mm^3^. She had no documented history of CD4 counts less than 200 cells/mm^3^ or other AIDS-defining conditions. 

Physical examination revealed a female patient in mild distress, without evidence of plethora. The patient had mild tachypnea but normal chest wall motion. She was sitting comfortably in a gurney with the head of the bed at approximately 45 degrees. She was speaking in full sentences and had normal oxygen saturation on room air. Heart and lung sounds were documented as normal. The patient’s abdomen was soft and non-tender without any masses. Initial vital signs were notable for a blood pressure of 100/62 mmHg, a heart rate of 97 beats/min, a temperature of 36.8°C. Initial laboratory data was noteworthy for a white blood cell count of 13,200 cells/mcL (segmented neutrophils of 81%), hemoglobin of 12.7 grams/L, a hematocrit of 38%, and a platelet count of 480,000 cells/mcL. Chemistry and coagulation panels were without significant abnormalities. To evaluate the patient’s chest pain, a chest radiograph and electrocardiogram were ordered. The electrocardiogram was unremarkable, with a normal sinus rhythm and no ST-T changes. Chest radiograph was notable for a large right suprahilar mass and a 2 cm pulmonary nodule.

**Figure 1:**
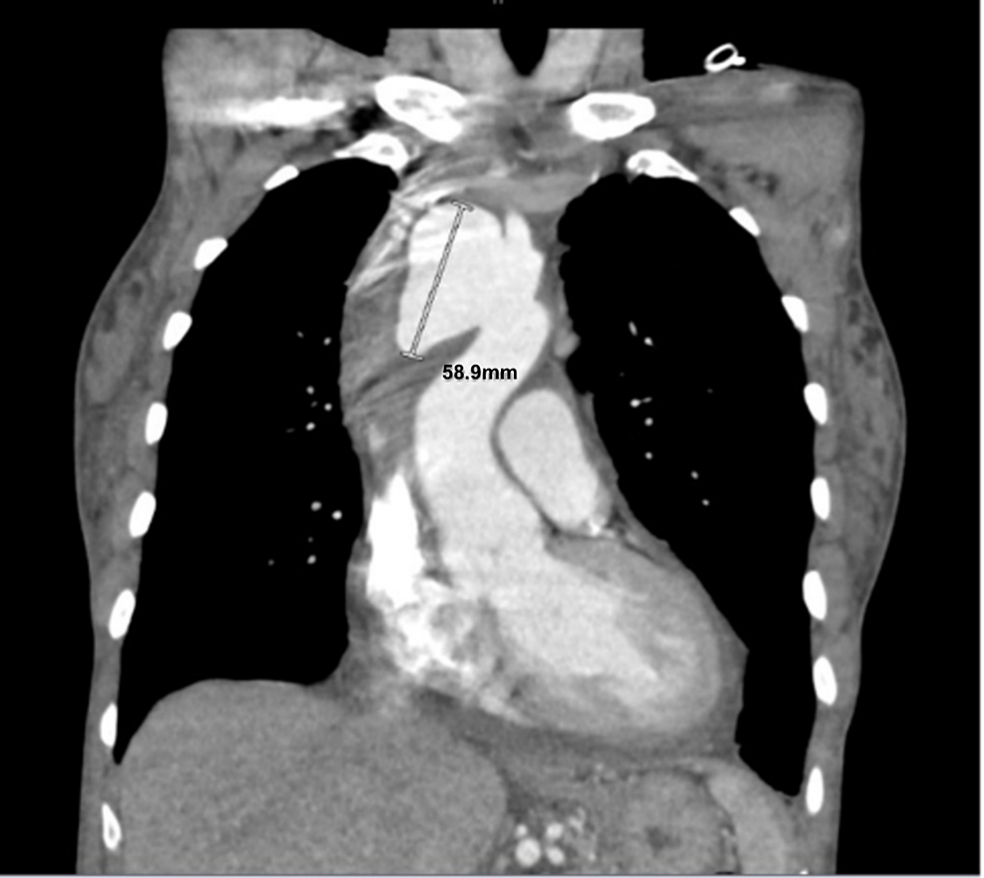
CT thorax demonstrating innominate artery aneurysm

For further characterization of the suprahilar mass, a CT scan of the thorax with IV contrast was obtained. The CT thorax demonstrated an eccentric lobulated saccular aneurysm arising from the origin of the brachiocephalic artery measuring 5.9 x 4.7 x 3.2 cm with adjacent mediastinal hemorrhage, free fluid, and a small amount of intermediate density pericardial fluid. The differential diagnosis offered by the radiologists included a mycotic aneurysm given the patient’s established HIV-positive status and a lack of imaging evidence to suggest significant atherosclerotic disease (Figure 1). Based on the CT findings, a cardiothoracic surgical consult was placed. 

**Figure 2:**
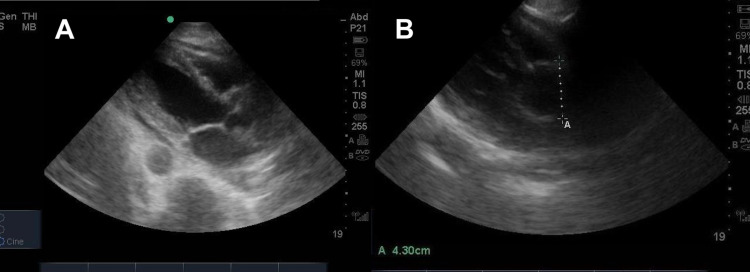
2A Bedside echocardiogram demonstrating pericardial effusion without evidence of tamponade 2B Bedside echocardiogram demonstrating widening of the aortic root measuring 4.3 cm in diameter

**Figure 3:**
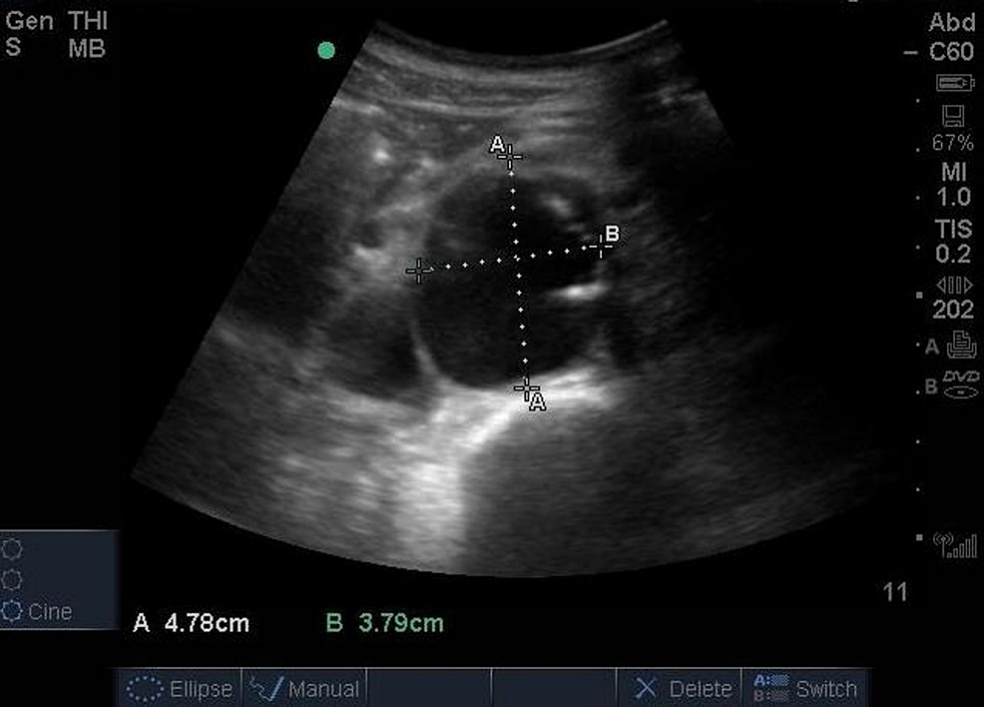
Ultrasound demonstrating abdominal aortic aneurysm measuring 4.78 cm X 3.79 cm on transverse view

Before the patient’s evaluation by cardiothoracic surgery, a PoCUS was performed in the ED to assess for cardiac tamponade and to image the abdominal aorta, which was not evaluated on the CT thorax. A bedside echocardiogram showed widening of the aortic root and a pericardial effusion without evidence of tamponade (Figure 2A, 2B). PoCUS of the abdominal aorta revealed an additional finding of a 4.8 x 3.8 cm infrarenal abdominal aortic aneurysm (AAA) (Figure 3 and Video 1). 

**Video 1:** Ultrasound demonstrating abdominal aortic aneurysm on transverse view

**Figure 4:**
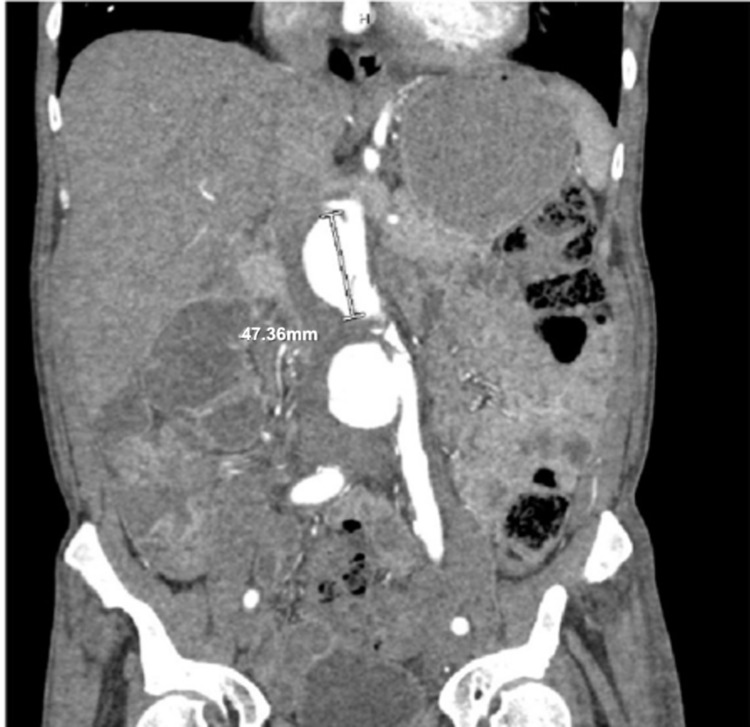
CT angiography abdomen demonstrating multiple infrarenal abdominal aortic aneurysms

The cardiothoracic surgery team was notified of the newly identified AAA seen on PoCUS, and they subsequently ordered a CT angiogram of the abdominal aorta with bilateral iliofemoral runoff. CT angiography demonstrated a large, lobulated infrarenal saccular AAA measuring 2.8 x 2.7 x 4.7 cm at the level of second and third lumbar vertebra, a saccular AAA measuring 4.1 x 4.1 x 3.5 cm at the fourth lumbar vertebra level, and dilation of the right common iliac artery to 1.7 cm (Figure 4). Based on the imaging findings from PoCUS and CT studies, a consult to the vascular surgery service was placed for possible surgical intervention for the patient’s intra-abdominal vascular pathology.

Based on the patient’s worsening clinical course, evidenced by increasing tachycardia and tachypnea, and imaging results, a decision was made to take the patient for immediate intervention for her pericardial effusion and thoracic, vascular abnormalities. Operative findings were significant for a purulent pericardial effusion, a thick rind covering the ascending aorta and aortic arch, and an innominate aneurysm. Pathology reports from surgical specimens showed necrotic vascular tissue with abscess formation, consistent with a mycotic aneurysm. Microbiology studies of the diseased aortic tissue identified *Haemophilus Influenzae* as the causative organism. 

Once medically optimized after her emergent thoracic surgery, the patient was taken back to the operating room by vascular surgery for abdominal aorto-bi-iliac bypass surgery with a cryograft. Operative findings were notable for contained rupture of the saccular abdominal aortic aneurysms and right common iliac artery aneurysm. Pathology and microbiology studies were again consistent with mycotic changes. The patient was continued on intravenous ceftriaxone and high-dose oral amoxicillin for the duration of her 21-day hospital course. Approximately one month after her presentation in the ED, during a routine one-week outpatient follow-up, the patient reported feeling well.

## Discussion

This case report of multiple mycotic aneurysms highlights the usefulness of PoCUS as an invaluable clinical tool that can aid in the diagnosis of life-threatening pathology in the ED [12]. Other case reports have described the diagnosis of multiple mycotic aneurysms by both ultrasound and CT imaging [2,13]. The present case report is notable in that the patient’s abdominal aortic pathology was first identified on PoCUS in the ED, which significantly changed how she was managed during her hospitalization. 

There are likely multiple mechanisms by which mycotic aneurysms arise in HIV+ patients. These include inflammation of the vasculature related to the virus, HAART, secondary opportunistic infections, or a combination of these factors [5,14,15]. Indeed, HIV-related aneurysms have been described as a possible distinct pathology resulting from an inflammatory response within large vessel walls, resulting in weakening of the vessel wall and subsequent aneurysm formation [9]. Multiple case series describe histopathology of mycotic aneurysms from HIV patients with no evidence of bacterial infection [1,2,13]. Conversely, other case reports detailing mycotic aneurysms in similar patients demonstrate numerous organisms, including *Treponema spp., Staphylococcus spp., Streptococcus spp., Enterococcus spp., Mycoplasma tuberculosis, Haemophilus influenzae *[9]. Similar to our patient, mycotic aneurysms caused by *Haemophilus influenzae* are often multiple and found in atypical locations from those seen in other conditions that lead to aneurysmal changes in the aorta and other large vessels (e.g., Marfan syndrome, Ehlers-Danlos syndrome, and hypertension) [1,9].

Given the high rate of mortality associated with aortic rupture, early diagnosis of infected aortic aneurysms is critical [7,16,17]. However, patient presentation and physical findings are usually nonspecific. The first symptoms may result from aneurysm expansion or rupture, resulting in diagnosis late in the disease course [18]. As such, bedside imaging modalities like PoCUS are a useful diagnostic tool for ruling out this potentially deadly pathology promptly.

PoCUS is a quick, portable, easily accessible, non-invasive exam, which can be performed at the bedside on unstable patients [12,19]. Unlike CT or angiography, it does not expose patients to ionizing radiation nor risk the patient decompensating outside of the ED [19]. While ultrasound is operator-dependent and visualization of the abdominal aorta can be limited by several patient factors (i.e., body habitus and overlying bowel gas), a systematic review and meta-analysis have shown that ED PoCUS has a sensitivity of 99% and a specificity of 98% for the detection of AAA [20-23]. Additionally, transthoracic echocardiography has demonstrated equivalency with transesophageal echocardiography and CT angiography for ascending aorta measurement, making it useful for ruling out thoracic vascular emergencies [24-26].

While ultrasonography can diagnose an aneurysm, even in atypical locations or with uncommon morphologies, it lacks a proven ability to detect infection. [21,27-30]. Although CT angiography is considered the method of choice for diagnosing mycotic aortic aneurysms, in one published case series, nearly 18% of cases were initially diagnosed by ultrasound [28]. Ultrasonography findings for mycotic aneurysms may include saccular wall expansion with mural thickening [21,30]. PoCUS imaging of mycotic peripheral vasculature aneurysm may depict a well-circumscribed, hypoechoic lesion adjacent to the main artery [21]. Larger mycotic aneurysms can show turbulent blood flow, with the classic “yin-yang” sign on color doppler imaging [21,30,31]. Eccentric or concentric periarterial hematoma and infected soft tissue can appear as a heterogeneous rind of variable echogenicity encircling the mycotic aneurysm lumen [21]. The use of PoCUS to identify both thoracic and abdominal aortic pathology in patients with a mycotic aneurysm may aid in the disposition of patients to the operating room for the appropriate surgical intervention [32,33].

Mycotic aneurysms should always be suspected in immunocompromised patients and those with independent risk factors arterial aneurysms (i.e., smoking, cocaine use, hypertension, intravenous drug use) [34]. Our case is an example of multiple aneurysms arising in an HIV+ patient with a history of polysubstance abuse where PoCUS guided the rapid diagnosis of additional mycotic aneurysms not captured on initial CT imaging. 

## Conclusions

PoCUS is an efficient diagnostic tool for early detection of ascending and abdominal aortic aneurysms, as well as pericardial effusions. It can be used for serial examinations and identify opportunities for intervention in critically ill patients. Our patient’s case hinged on detecting her AAA using PoCUS in the ED. Initial surgical intervention for ascending aortic repair was followed by communication of the abdominal ultrasound findings and later confirmed by CT angiogram. This led to a second surgery for the mycotic AAA once the patient was medically optimized. Thus, this case reinforces how ED PoCUS can guide more extensive diagnostic imaging and surgical intervention for patients at high risk of multiple aneurysms such as those with HIV or other immunocompromised states.
